# Comparative Analysis of Highly Purified Sericin and Waste-Derived Sericin: Implications for Biomedical Applications

**DOI:** 10.3390/biomimetics10060387

**Published:** 2025-06-11

**Authors:** Federica Paladini, Fabiana D’Urso, Angelica Panico, Carmen Lanzillotti, Francesco Broccolo, Mauro Pollini

**Affiliations:** 1 Department of Experimental Medicine, University of Salento, Via Monteroni, 73100 Lecce, Italy; fabiana.durso@unisalento.it (F.D.); francesco.broccolo@unisalento.it (F.B.); 2 Caresilk S.r.l.s., Via Monteroni c/o Technological District DHITECH, 73100 Lecce, Italy; angelica.panico@caresilk.it (A.P.); carmen.lanzillotti@caresilk.it (C.L.)

**Keywords:** sericin, silk protein, protein purification, waste valorization, biomaterials, tissue engineering

## Abstract

Sericin, a natural glycoprotein constituting 20–30% of the silk cocoon, has emerged as a promising biomaterial due to its excellent biological properties, including biocompatibility, antioxidant properties and potential applications in regenerative medicine. The quality and the features of sericin are strongly dependent on the extraction and purification methods, which can employ mild conditions to preserve the molecular integrity of the protein or recovery techniques from waste streams produced during the industrial degumming processes. The silk industry prioritizes fiber yield over protein preservation, so often harsh alkaline conditions at high temperatures are adopted. These divergent approaches result in fundamentally different products with distinct molecular characteristics and functional capabilities. This review comprehensively examines the current technological approaches for sericin extraction techniques and for its recovery from textile industry waste, focusing on how these aspects affect the biological properties of the protein and the potential applications.

## 1. Introduction

Sericulture, which has contributed to the worldwide textile industry, is a complex and ancient trade involving silkworm rearing, the harvesting of cocoons and the extraction of silk fibers. According to the Food and Agriculture Organization (FAO), the global production of silk fibers has been estimated to be around 175,000 tons in 2020 and was valued at approximately USD 7 billion. Silk fiber is an ancient textile material dating back to the 27th century BC in China [1]. It is produced by different types of arthropods, such as spiders, flies, mites, scorpions, bees and silkworms, including mulberry (*Bombycidae*) and non-mulberry silkworms (*Saturniidae*) [2,3]. Among them, mulberry silk produced by *Bombyx mori* is generally preferred because of its commercial feasibility compared to other silk types [2].

Beyond its role as a textile material, silk is also a biomaterial mainly composed of two proteins, namely fibroin and sericin, which have attracted significant attention in biomedical research due to their unique combination of mechanical properties and biological functionality [4,5,6]. While fibroin has been extensively studied, its cocoon-mate sericin has only recently gained recognition as a valuable biomaterial in its own right. Sericin, the glue-like protein that envelops fibroin fiber in silk cocoons, constitutes approximately 20–30% of the total cocoon weight and plays crucial roles in the formation of fiber and protection [7,8,9,10]. Historically, sericin has been considered as a waste product, often discarded during the degumming process in silk production. However, growing evidence of its biological activities, such as its remarkable biocompatibility, biodegradability, antioxidant and cell proliferative properties, has spurred an interest in sericin as an auspicious biomaterial for a wide range of applications [1,11]. These include wound dressings [12,13,14,15], drug delivery systems [12,16,17], tissue engineering applications [18,19] and cosmetic formulations [20,21]. The expanding applications have created a need to better understand how different processing methods affect the structure and functions of sericin. Moreover, the growing interest in more sustainable and environment-friendly industrial processes has addressed sericin as a renewable resource that can support circular economy approaches [1].

However, the quality and properties of sericin vary dramatically depending on its extraction and purification methods. Mild and optimized extraction conditions adopted to preserve molecular integrity, compared to industrial degumming processes which prioritize fiber yield over protein preservation, determine fundamentally different products with distinct molecular characteristics and functional capabilities.

This review provides a comprehensive analysis between highly purified sericin and waste-derived sericin, analyzing their structural differences, functional properties and potential applications. The implications of these differences for biological properties are also investigated, providing critical insights for optimizing sericin-based products.

## 2. Distinctive Features of Silk Sericin

Through millions of years of evolution, silkworms have developed highly specialized cocoon structures as protection for the developing pupa and for the precise control over moisture and gas exchange. This natural optimization process has resulted in silk proteins with an extraordinary combination of biological properties that modern biotechnology seeks to replicate and harness.

The remarkable features of sericin exemplify nature’s sophisticated approach to multifunctional biomaterial designs, making it a compelling subject for biomimetic research and applications. By understanding and mimicking these natural properties, researchers can develop innovative extraction and processing methods that preserve the protein’s native functionality while adapting it for specific biomedical applications, thus bridging the gap between biological inspiration and technological innovation.

Secreted in the mid-region of the silk gland, silk sericin (SS) is a globular protein characterized by the presence of 18 amino acids, with predominantly serine (40%), glycine (16%), glutamic acid, aspartic acid, threonine and tyrosine [11]. The molecular weight ranges from 24 to 400 kDa, with three major fractions isolated from the cocoon corresponding to 400, 250 and 150 kDa, which are related to the middle, anterior and posterior part of the middle silk gland, respectively [22]. SS contains polar side chains with various functional groups, such as amine, hydroxyl and carboxyl groups, which can enhance the structural stability by promoting physical or chemical interactions with other polymers through blending and crosslinking methods [23].

Solubility is a feature related to the amorphous and crystalline structure of sericin [24]. The amorphous regions with a random coil structure correspond to the readily soluble sericin, while the crystalline regions formed by the β-sheet structure are more difficult to dissolve [24]. In a partially unfolded state, the beta-sheet and random coil represent 35% and 63% of the random coil of the molecular structure, respectively [23,25]. Based on its solubility and molecular weight, different fractions of sericin have been identified. The most soluble fraction is named Sericin A, which is located in the uppermost stratum of the cocoon and comprises serine, threonine, glycine and aspartic acid as its most abundant amino acids. The other fractions, Sericin B and Sericin C, with lower solubility are present in the intermediate and in the inner stratum [8]. With respect to the classification of sericin on the basis of its molecular weight, Sericin A, M and P have been distinguished, following the site of biosynthesis in the middle area of the silk gland [8].

**Figure 1:**
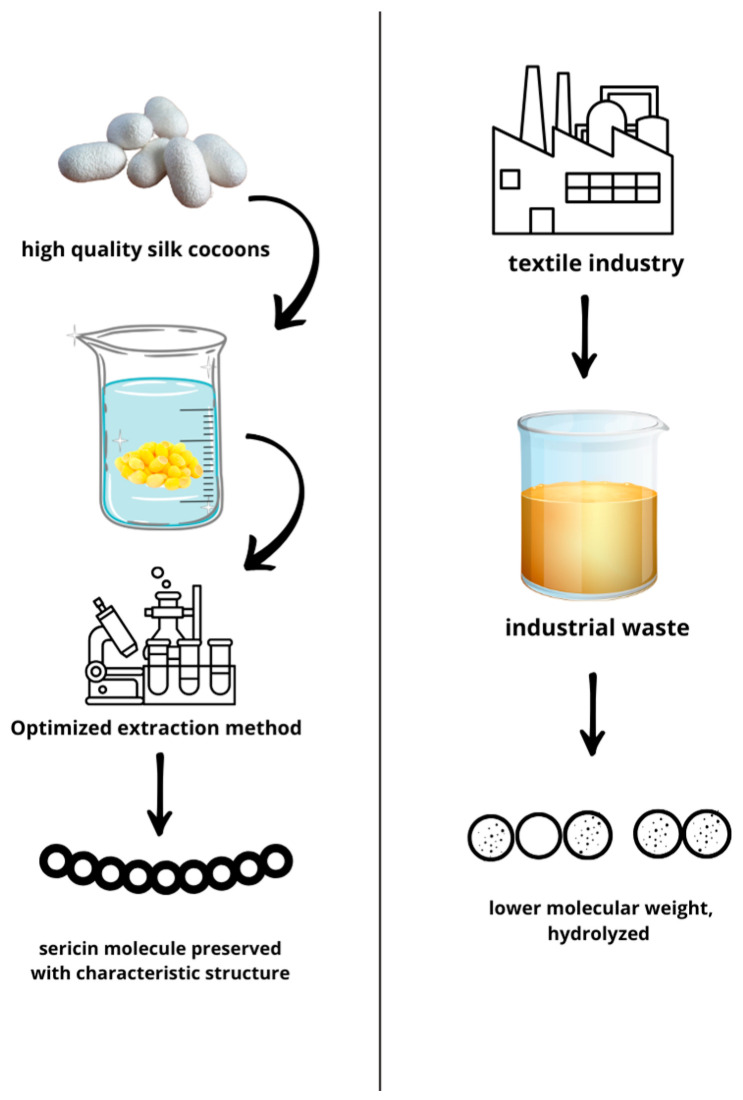
Pure sericin with a high molecular weight obtained by an optimized extraction protocol vs. waste-recovered sericin with a lower molecular weight and contaminants.

The silk cocoon is the preferred source for obtaining pure sericin, as it has only been dried for preservation purposes without being exposed to any thermo-chemical process. Silk cocoon degumming is the wet processing approach to remove sericin from raw silk and to separate sericin from fibroin [26,27]. To achieve optimal silk degumming and sericin extractions, various methods are employed, each with its advantages and drawbacks [24]. Indeed, various methods for the extraction of sericin have been reported to have a role in the effective anti-tyrosinase potential of sericin due to the numerous serine, asparagine and threonine residues acting as chelators [28]. The molecular weight of SS extracted by cocoons and recovered by wastewater is significantly influenced by the method of extraction and affects the domains in which sericin could be used (Figure 1).

Different molecular weights (MWs) of sericin exhibit varying characteristics [29]. For instance, a high MW demonstrates an excellent antioxidant capacity [30] and antibacterial properties and a higher viscosity, gel–sol transition temperature and mechanical strength. Moreover, a higher MW corresponds to a higher number of β-sheet structures, which is associated with a stronger osteogenic activity. The extension rate of the sericin film is also improved when compared with lower MWs [3]. A thorough understanding of the SS molecular mechanism and extraction methods can enhance its applications.

## 3. Sources for Sericin Extraction and Recovery 

SS has been discarded as a byproduct of the sericulture industry for thousands of years, receiving significantly less attention than fibroin [19]. More recently, the great potential of sericin in biomedical research and applications has been recognized and many advances have been made in the development of sericin-based biomaterials for tissue engineering and regenerative medicine [19]. Indeed, biodegradability, bioavailability, a pH- and temperature-responsive capability, a natural photoluminescence and antioxidant properties have been demonstrated in sericin, which has also been addressed as GRAS and FDA-safe food additives [27].

The principle of silk degumming is based on the different solubility between insoluble fibroin and highly hydrophilic soluble sericin. Silk degumming can be achieved by physical methods, such as boiling and high-temperature and high-pressure (HTHP) methods, chemical methods, by using degumming agents, and a combination of both physical and chemical methods [31]. In silk production, the initial phase of the process consists of cooking cocoons in hot water to kill the silkworms. Then, after unwinding the silk fibers, reeling and weaving are performed. After the production of the silk yarns, the degumming process is applied for removing sericin in order to prepare the yarns for dyeing [32]. As one of the most abundant byproducts of textile manufacturers [31], discarded in the wastewater after the degumming, sericin intensifies the contamination of the environment through the increase in the chemical oxygen demand (COD) and biological oxygen demand (BOD) [31]. A total production of 109,111 tons of silk has been estimated in the world in 2019 (International Sericultural Commission, 2021) [33], and 50,000 tons of sericin have been generated yearly worldwide [31]. The large quantities of alkaline wastewater containing sericin generated in industrial silk production can cause severe environmental pollution, and this is a huge waste of biological resources that are difficult to recover [29,34]. Thus, recovering and recycling this protein represents an environmental and economic challenge [31] which can provide economic and environmental benefits, although recovering a resource from wastewater may require additional processes compared to treatment technologies, thus causing additional impacts on the environment [33].

Moreover, it is a big challenge to obtain sericin protein that is high-quality, without degradation and denaturation, on a large or industrial scale [19]. A comparison between sericin derived by the cocoon cooking process and by the silk degumming process has demonstrated different distributions of molecular weights, corresponding to 10 to 200 kDa in the first case and to 100–120 kDa in the second scenario [32]. Moreover, while cocoon cooking waters contain the protein, silk degumming wastewaters also contain soap, bleach and softening agents in addition to sericin [35]. Degumming agents, such as soap, alkali, synthetic detergents or organic acid, are commonly used in the degumming process to break the peptide linkage of the amino acid into small molecules in order to hydrolyze and dissolve sericin in water [31,34].

This method successfully removes sericin from cocoons, allowing for the recovery of clean and isolated fibroin for applications in the textile industry. However, the recovered sericin is highly degraded, reducing its molecular weight and losing some functional properties. In addition, the separation of the soap and sericin is very complex, and traces of soap in the protein are difficult to remove, thus limiting its use for biomedical and pharmaceutical purposes [36]. According to the purpose of use, the price of dry sericin ranges from EUR 40/kg (for ISO9001 Certified Sericin) [33] up to about EUR 200/g (Sigma-Aldrich, St. Louis, MO, USA, Sericin *Bombyx mori*), reflecting the physical properties and purity [37].

In response to sustainability concerns and circular economy principles, numerous methods have been developed to recover sericin from textile industry wastewater. However, the biological properties of sericin derived from these different sources—directly extracted from cocoons versus recovered from textile wastewater—exhibit notable variations that significantly impact their potential applications.

## 4. An Overview of the Degumming Processes

The extraction and purification of SS is a crucial step that determines the quality, structural integrity and application potential of the final product. Over the years, several degumming methods have been developed to remove the sericin coating from fibroin fibers, each with specific mechanisms, efficiencies and impacts on the protein structure. These approaches can be broadly categorized into chemical, thermal, enzymatic and novel green technologies. After degumming, further purification methods can be applied to isolate pure sericin. This section aims to provide a comprehensive description of each method, highlighting the advantages and limitations and their influence on the physicochemical and biological properties of sericin.

Among the most conventional methods, boiling in Marseille soap derived from olive oil has been used for over two centuries for its high effectiveness in removing sericin due to the high degree of the hydrolysis of the protein, which results in molecular weight (MW) distributions typically ranging between 20 and 43 kDa [38]. The separation of sericin from the soap is very difficult, so the recovery of sericin requires a comprehensive purification [39]. In addition, the high costs of Marseille soap, along with the environmental concerns associated with water quality issues, have moved the attention to other alternatives such as degumming with heat or alkalis [12,39]. Chemical degumming, one of the most traditional methods, typically employs alkaline substances, such as sodium carbonate or sodium hydroxide, or acidic agents like citric and tartaric acids. These reagents break peptide bonds in the sericin structure, enabling hydrolysis and solubilization and often leading to a significant protein degradation released in an alkaline or acid solution [39].

As a result, the recovered sericin displays lower molecular weights and altered functional properties, which can limit its use in biomedical fields. Furthermore, the separation of sericin from chemical agents requires extensive purification and poses environmental concerns due to wastewater generation [36,40].

To reduce this significant degradation of sericin, urea or urea combined with 2-mercaptoethanol extraction methods has been adopted. The results demonstrated a reduction in protein damages and a preservation of a broad MW range (10–225 kDa), compared with the acid- and alkali-degraded sericin which exhibited clear bands within the ranges of 50–150 kDa and 15–75 kDa, respectively [36,40].

Dialysis or extensive washing steps required to reduce cytotoxicity can make the process less eco-friendly and more expensive, so it is important to optimize both the extraction process and robust purification strategies [36,40].

Alternatively, enzymatic approaches based on the use of trypsin, papain and bacterial enzymes have emerged thanks to their efficiency in energy consumption [31,34]. In enzymatic degumming, the enzyme action selectively attacks specific parts of the silk, removing sericin by hydrolysis. The type and concentration of enzymes, along with the duration of the reaction, significantly affect the process kinetics [12,39,41]. Enzymatic degumming using papain, trypsin or proteases from microbial sources offers a selective and mild approach to sericin removal and reduced structural damages [31]. This method can retain bioactive peptide sequences and reduce denaturation, but it is less suitable for large-scale applications due to high costs, process complexity and longer treatment times [31,34,39].

Thermal extraction, which consists of boiling cocoons in hot water ranging between 80 °C and 100 °C under atmospheric pressure or at an increased pressure, offers the advantage of ensuring the absence of impurities and the possibility of using recovered sericin without dialysis. Although the potential degradation of sericin can occur in thermal extraction depending on the temperature, pressure and prolonged heating, sericin maintains its excellent properties [42]. Indeed, high-temperature autoclaving at 1 kgf/cm^2^ (kilogram-force per square centimeter) for 40 min has been shown to effectively extract sericin with molecular weights ranging from 20 to 400 kDa, with minimal denaturation after the ethanol or freeze–thaw precipitation [43], making it suitable for biomedical use. This method is also highly appreciated for its low chemical processing and is considered environmentally friendly [42].

Due to the growing interest in sustainability and green chemistry, the development of innovative extraction techniques has been recently addressed at minimizing environmental impacts and reducing the water consumption during extraction. These include methods based on infrared heat, microwaves, steam treatments, carbon dioxide supercritical fluid and ultrasonication. In particular, microwave- and infrared-assisted degumming eliminate or reduce the need for chemical agents, achieving a MW retention > 100 kDa and improving sustainability profiles [39,44]. These methods are characterized by fast and uniform heating, showing potential in preserving the structural and functional integrity of sericin and in achieving the high-purity and cost-effective extraction of sericin, with minimal denaturation and degradation. Ultrasonic-assisted extraction improves the sericin release through cavitation, while supercritical CO_2_ offers a solvent-free approach with high purity, though it remains technologically complex and expensive for routine use [39].

In recovery methods from textile wastewater, where sericin co-exists with soap/detergents and other impurities, membrane processes are necessary for separating sericin from the chemicals and contamination. Membranes also concentrate sericin, thus reducing the chemical consumption post-precipitation [45]. Among membrane technologies, ultrafiltration and nanofiltration have emerged as promising alternatives for achieving recovery rates higher than 80% with a higher purity of sericin while minimizing the degradation of the protein [39]. The combination of nanofiltration and ultrafiltration has been proposed for recovering higher molecular weights through ultrafiltration, while rejecting low molecular weights through nanofiltration [46].

Precipitation methods, including acidulation precipitation, chemical coagulation, organic solvent precipitation and salting out, can also be adopted to separate the protein from the solution. The methods involve the addition of a precipitating agent (e.g., trichloroacetic acid, calcium chloride and ammonium sulfate) or freezing/thawing techniques [39]. The molecular weight distribution of sericin extracted by autoclaving and then concentrated via the precipitation by ethanol and by freezing/thawing was compared by Da Silva et al. [43]. In particular, the ethanol precipitation method involved pouring SS over the same volume of ethanol and then maintaining the mixture at 4 °C for 24 h to improve the precipitation and to reduce the denaturation of the protein. In the precipitation by freezing/thawing, on the other hand, the SS was frozen in a conventional freezer for 24 h and then it was thawed at room temperature. The results of size exclusion chromatography showed that the degumming process in the autoclave at 1 kgf/cm^2^ for 40 min was effective in extracting sericin with high molecular weights ranging between 20 kDa and 400 kDa and that these values were confirmed after both of the precipitation methods [43]. The addition of ethanol to fibroin determines a reduction in sericin solubility, and the separation from wastewater can then be achieved through centrifugation. The conformation changes in the course of the ethanol precipitation were studied by Wu et al. by circular a dichroism analysis. Although the major conformation of sericin was a random coil, the treatment with ethanol induced the packing of molecular chains and the formation of β-sheet conformations. The data collected by the authors also denoted a potential degradation of sericin into lower-molecular-weight species during the preparation [47]. Mira et al. demonstrated a high impact of the concentration method on the structural conformation of the sericin samples. The authors addressed the freeze–thaw concentration process as a method providing structural and thermal stability and generating less protein degradation, with more crystalline and stable structures depending on the nucleation of ice crystals, which consequently generates the agglomeration of long peptide chains [48].

**Figure 2:**
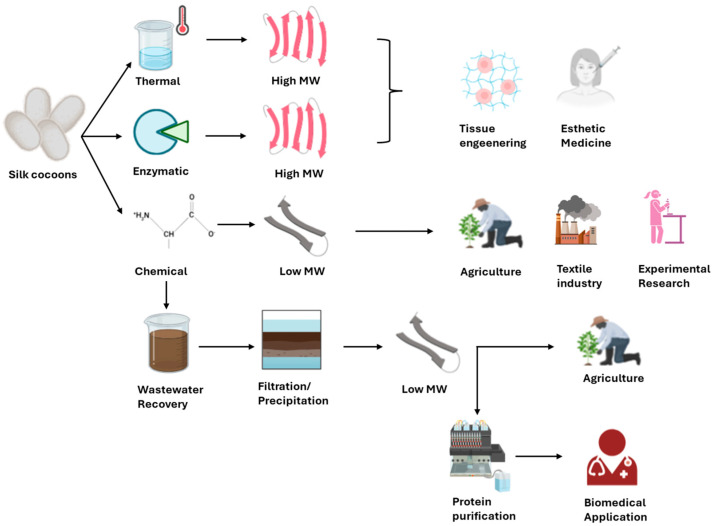
High-purity sericin can be obtained from silk cocoons through thermal and enzymatic extractions and it can be applied in tissue engineering and esthetic medicine fields. Less pure forms of sericin can be obtained by chemical extraction for application in agriculture field, textile industry and experimental research. Sericin derived from wastewater recovery can be applied for some biomedical applications only if properly purified.

The comparative analysis of the extraction methods is necessary to guide researchers and industry professionals in choosing the most appropriate approach based on technical and practical requirements. Given the wide range of available techniques, the selection of a suitable degumming method must consider the intended application of the final product. While high-purity sericin is essential for medical and cosmetic applications, less pure forms may be acceptable in agriculture or textile finishing (Figure 2).

## 5. The Effect of Different Degumming Methods on the Biological Properties of Sericin

Degumming methods influence not only the yield and structure of sericin but also its biological performance. Numerous studies have demonstrated that the antioxidant capacity, cell adhesion and biocompatibility of sericin are closely related to its molecular weight, amino acid profile and secondary structure [19,39]. In particular, as demonstrated by Liu et al., high-MW sericin (200–400 kDa) exhibits an enhanced antioxidant activity, supports fibroblast proliferation and promotes osteogenic differentiation [19,40], whereas low-MW sericin (<20 kDa), which is common in waste-derived or chemically treated samples, shows a reduced bioactivity and poor cell adhesion [19,47].

High-molecular-weight sericin, typically obtained through gentle thermal extractions, retains functional amino acid residues, such as serine, threonine and tyrosine, which contribute significantly to free radical scavenging. Additionally, preserved β-sheet conformations enhance cellular interactions and support collagen synthesis and the proliferation of fibroblasts [12,40,49]. The interactions of SS films with a molecular weight of 400 kDa, sericin M, evaluated with human skin fibroblasts have shown an increase to 250% of cells after an incubation for 72 h, which increased with respect to the control [26].

In contrast, harsh chemical treatments often fragment sericin chains, reduce the molecular weight and disrupt secondary structures, resulting in diminished bioactivity and increased cytotoxicity, especially when chemical residues remain [40,47]. Although allowing a MW of 10 to >225 kDa, without a rigorous purification, the residual urea in the urea-based protocol is known to be cytotoxic and to cause a low collagen production, particularly at concentrations higher than 100 μg/mL [36,40]. Thermal methods, in particular, preserve the MW and protein conformation, resulting in sericin with a cell viability above 90% in fibroblast assays [40], while alkaline-degummed sericin shows a <60% viability, along with structural denaturation and β-sheet loss [36]. Wang et al. used high temperatures (160, 200 and 220 °C) to extract sericin hydrothermally, demonstrating that the hydrothermal treatment performed at 220 °C was effective in extracting sericin with a yield above 80%, high antioxidant ability, strong tyrosinase inhibition and preservation of native β-sheet structures [49].

FTIR analyses show that β-sheet-rich sericin (common in thermal extractions) promotes mechanical stability and cell attachment, while random-coil-dominant sericin (often from alkaline or enzymatic processes) exhibits a lower performance [47].

Minimizing chemical degradation is essential for enhancing the cell growth and attachment, primarily due to the spatial arrangement of methionine and cysteine residues. Additionally, the amino acid composition of sericin plays a crucial role in its biological properties and performance as a biomaterial. For instance, sericin with higher concentrations of serine and threonine demonstrates improved antioxidant and photoprotective activities [39], and the repetitive sequence of serine amino acids in SS has been associated with an increased cell adhesion activity [26]. The arrangement of sericin’s amino acids in terms of aggregation, β-sheet formation and β-turns can influence cell behavior [39]. As a foreign protein, the recognition of sericin by the receptors of the immune cells is influenced by the protein conformation and particularly by β-sheet structures [19]. Along with hydrolysis and the formation of low molecular weights, the degumming with alkalis and soaps also affects the conformation of the sericin, disrupting its secondary structure and suggesting the denaturation of the protein [36,38,50]. Wu et al. reported that the MW of sericin in wastewater could be as low as 6 kDa, possibly due to degradation [32,47]. These hydrolyzed protein losses affect functional properties, like the antioxidant capacity, gelling capacity and mechanical structure [30,50].

Another important challenge is to obtain a sericin free of chemicals. In the case of the acid and alkaline degumming solution, purification steps such as dialysis are necessary, thus making the process more expensive and less eco-friendly [26]. Moreover, large amounts of wastewater containing chemicals emphasize environmental issues [35,44], particularly in the case of the release of high quantities of soap and alkalis in the environment, which makes the process highly polluting [26]. While urea-based extraction methods pose environmental challenges due to the toxicity of urea and the hazardous waste produced [39], thermal extraction by using hot distilled water has low environmental and chemical impacts, allowing for the production of sericin free from impurities and the preservation of its remarkable properties [36,39], making this method economically feasible and scalable and compliant with industrial applications [36,39].

These findings underscore the importance of matching the extraction strategy to the intended application. Table 1 summarizes the most relevant aspects for comparing advantages and disadvantages of the different methods for obtaining silk sericin. For high-purity biomedical use (e.g., scaffolds and drug delivery), methods that preserve the MW and β-sheet content while avoiding contaminants—such as thermal- and membrane-assisted techniques—are preferable. For applications where purity demands are lower (e.g., cosmetics or agriculture), chemically extracted sericin may still be appropriate if adequately purified.

## 6. Potential Applications of Highly Purified Sericin vs. Waste-Derived Sericin

**Figure 3:**
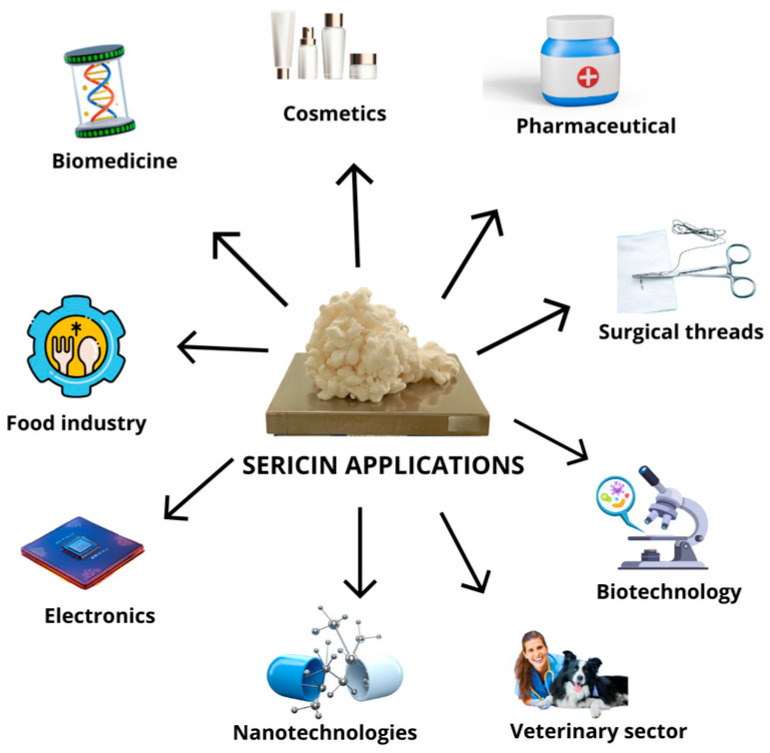
Examples of application of highly purified silk sericin obtained by optimized extraction protocols from cocoons.

In recent years, silk sericin has gained increasing commercial attention across diverse sectors, from high-end biomedical and cosmetic products to low-cost industrial uses. The market positioning of sericin largely depends on its purity and molecular integrity and structure. These biological properties of sericin vary significantly depending on whether the protein is directly extracted from cocoons or recovered from textile industry waste. Highly purified sericin, typically extracted through optimized water-based or enzymatic processes, commands a premium value due to its superior biological properties, making it suitable for advanced medical applications, such as tissue engineering scaffolds, wound dressings, cosmetic formulations, etc., (Figure 3) [8,9,10,20].

From a biochemical perspective, the preservation of sericin’s native structure through gentle extraction protocols appears crucial for maintaining its valuable biological activities. The intact molecular weight distribution and balanced secondary structure composition of purified sericin correlate directly with its superior mechanical properties, antioxidant capacity and cellular compatibility. These findings suggest that sericin’s functional motifs are particularly sensitive to thermal and chemical degradation, emphasizing the need for precisely controlled processing conditions in biomedical applications.

In contrast, waste-derived sericin, recovered from textile industry wastewater, is structurally degraded, resulting in lower molecular weights, altered structural conformations and low bioactivities. This often limits its use to less demanding applications, for example, as a biostimulant in agriculture, an additive in animal feed, or a raw ingredient in industrial formulations [27,28,33,47].

Understanding these distinct market trajectories provides important context for optimizing extraction strategies and guiding application-specific sericin development.

The stark differences between highly purified and waste-derived sericin also underscore how operational parameters determine the biological performances and have profound implications for the development of sericin-based technologies across multiple sectors, with a particular relevance for production scale-up. While the laboratory-scale purification yields a superior material, the economic and environmental benefits of valorizing textile waste cannot be overlooked. This creates an interesting challenge for process engineers for properly adapting the principles of gentle extraction and advanced purification to industrial-scale operations without compromising cost-effectiveness. On the other hand, while sericin recovery from textile wastewater aligns with circular economy principles by transforming an industrial byproduct into a valuable material, it is important to recognize that this process can introduce new environmental burdens. High-purity recovery often requires energy-intensive processes, such as ultrafiltration, ethanol precipitation and multiple purification steps to eliminate surfactants and alkaline residues [31,35,45]. These additional stages can increase both the energy demand and environmental footprint of the overall process, particularly in terms of water consumption and chemical waste generation [33,36]. Therefore, the sustainability of sericin recovery must be evaluated through a life cycle perspective, balancing the benefits of waste valorization against the inputs required for purification [33]. The environmental advantages of sericin recovery are maximized when high-impact purification steps are avoided, either by targeting applications that tolerate lower purity levels or by developing integrated, resource-efficient recovery platforms [29,35]. Moreover, while waste-derived sericin may present a cost-effective and sustainable material for low-risk applications such as agriculture, its use in environmental settings requires a careful consideration of residual toxicity. Current purification methods may not fully eliminate detergents, alkalis, softeners or even trace heavy metals introduced during the textile degumming process. Although some studies have reported positive effects of sericin on seed germination and plant growth, there are limited empirical data on the ecotoxicological profile of incompletely purified sericin [33]. Residual surfactants, in particular, can alter the soil microbiota or disrupt plant nutrient uptake. Moreover, life cycle assessments have highlighted that insufficient purification could potentially shift the environmental burden downstream. Therefore, before recommending waste-derived sericin for agricultural or ecological applications, further investigations of its environmental safety, contaminant bioaccumulation and soil/water interactions are essential. This remains an important gap in the literature and a priority for future research [33].

Another important consideration for product development is related to the stability of the material, potentially influencing packaging and distribution strategies. The remarkable shelf-life of purified sericin in a lyophilized form suggests that dry formulations may be preferable for commercial products. Several studies have demonstrated that lyophilized or freeze-dried sericin can retain its structural and functional integrity over extended periods. For example, Silva et al. reported that sericin powders maintained their antioxidant and antimicrobial activity after 12 months of storage at room temperature [36]. Similarly, Wu et al. observed that sericin extracted from industrial wastewater and stored in a powder form under low-humidity conditions exhibited negligible molecular degradation over 6 months [47]. Moreover, Rueda Mira et al. confirmed that freeze–thaw-concentrated sericin showed a high thermal and structural stability, with minimal conformational changes during storage [48]. These findings support the feasibility of the long-term preservation of purified sericin, especially when dry-stabilized through appropriate techniques. Conversely, the limited stability of waste-derived sericin solutions may necessitate alternative formulation approaches or prompt-use applications.

From an application point of view, the performance gap between purified and waste-derived sericin suggests that these materials may naturally find different market niches.

Looking forward, the evolution of sericin applications will likely be shaped by two parallel developments: the continued refinement of purification technologies to enhance the yield and quality from one hand and innovative engineering approaches to adapt material properties for specific applications on the other hand. Future research should focus on optimizing recovery methods to better preserve the biological properties of waste-recovered sericin, potentially bridging the quality gap between the two sources while maintaining the environmental benefits of resource recovery. Future research should also focus on some key areas: (1) developing targeted extraction methods that preserve specific bioactive domains even in waste recovery processes; (2) establishing standardized quality assessment protocols specifically designed for waste-derived sericin; (3) optimizing purification techniques to remove process-specific contaminants; and (4) exploring synergistic combinations of waste-derived sericin with other biomaterials to enhance performance.

Furthermore, beyond optimizing extraction and recovery conditions, post-processing strategies can be functional in restoring or enhancing the performance of degraded or low-quality sericin, particularly sericin recovered from industrial waste streams. Among these approaches, chemical crosslinking using agents such as glutaraldehyde, genipin or EDC/NHS has been demonstrated to be effective in improving mechanical strength, reducing solubility and enhancing the stability of sericin-based films and scaffolds [20,23,40]. In polymer blending, sericin is combined with natural or synthetic polymers, like chitosan, alginate, gelatin or PVA, to create composite materials with a superior mechanical integrity and biological activity [4,20,39]. Surface modification techniques, including plasma treatments or the chemical grafting of bioactive molecules, have also been employed to improve biocompatibility and promote controlled interactions with cells or therapeutic agents [19,23], while nanostructuring and encapsulation strategies have been proposed to allow the incorporation of even low-molecular-weight sericin fractions into delivery systems, thus offering advantages in terms of stability, release control and targeting [17,25]. These approaches not only extend the usability of waste-derived sericin but also open promising avenues in fields such as cosmetics, agriculture and low-risk biomedical uses, including wound care, temporary barriers or topical formulations.

The optimal approach will likely involve the complementary use of both conventional extraction and waste recovery methods, with selections based on the careful matching of material properties with application requirements.

## 7. Conclusions and Future Outlook

This comprehensive review has examined technological approaches for sericin extraction and for its recovery from textile industry waste, evidencing that the conventional extraction yields higher-purity sericin with more predictable properties for biomedical applications, while waste recovery methods represent a sustainable alternative with adequate functional properties for many different purposes. However, the technological landscape for waste-derived sericin continues to evolve rapidly, with advances in separation, purification and characterization technologies progressively increasing. This analysis suggests that the future of sericin technology lies in developing a complementary portfolio of materials tailored to different performance requirements and market needs. By maintaining rigorous standards for medical-grade sericin while continuing to innovate in waste valorization, the field can maximize both the scientific and social impact of this versatile natural protein. By advancing our understanding of sericin structure–function relationships and refining production technologies, we can unlock the full potential of this remarkable natural protein for biomedical and biotechnological applications.

## Figures and Tables

**Table 1:** A comparative table showing the most relevant degumming methods to obtain sericin.

Degumming Method	Yield	Molecular Weight (MW)	Purity	Cytotoxicity	Environmental Impact	Post-Degumming Purification	Chemical Characteristics	Physical Characteristics	Biological Characteristics	Advantages	Disadvantages	References
Hot Water (Boiling/Autoclave)	High	20–400 kDa	High	Low	Low	Not necessary	No protein hydrolyzation; low chemical contamination	Maintains β-sheet and random coil balance	High antioxidant and cell compatibility	Eco-friendly, scalable, structure-preserving	-	[26,36,42,49]
Soap	Moderate–High	20-43 kDa	Low	Moderate	High (soap in wastewater)	Soap–sericin separation	Hydrolyzed protein	Disrupted β-sheet structure	Loss of antioxidant and gelling properties	Effective degumming	Poor purification, polluting	[12,38,39]
Alkaline	High	15–75 kDa	Low	Moderate–High	High	Dialysis	Hydrolyzed protein	Disrupts β-sheet structure	Limited bioactivity	Cost-effective degumming	Poor biofunctionality	[26,36,38,42,50]
Acidic	Moderate	50–150 kDa	Low	Moderate	High	Dialysis	Hydrolyzed protein	Structural instability	Limited bioactivity	Cost-effective degumming	Poor biofunctionality	[36,42]
Urea	High	10 to >225 kDa	Low	High	High	Dialysis—not always effective	Minimal hydrolysis	Maintains β-sheet and random coil structure	Cytotoxic	Minimizes degradation, well-defined MW	Strong cytotoxicity	[12,36,40,42]
Enzymatic	Variable	10–12 kDa	High	Low	Low–Moderate	Not necessary	Selective cleavage of peptide bonds	Limited degradation	Biocompatibility	Eco-friendly	Not scalable	[12,31,34,39,41]

## References

[1] Kabir M., Hasan M.K., Rafi M.N., Repon M.R., Islam T., Saha J., Rahman M. (2024). Biowaste Transformation to Functional Materials: Structural Properties, Extraction Methods, Applications, and Challenges of Silk Sericin. Select.

[2] Saad M., El-Samad L.M., Gomaa R.A., Augustyniak M., Hassan M.A. (2023). A comprehensive review of recent advances in silk sericin: Extraction approaches, structure, biochemical characterization, and biomedical applications. Int. J. Biol. Macromol..

[3] Yuan Y., Nasri M., Manayi A., Zhang J., Wu C., Jeon T.J., Kang L. (2024). Sericin coats of silk fibres, a degumming waste or future material?. Mater. Today Bio.

[4] Paladini F., Pollini M. (2022). Novel Approaches and Biomaterials for Bone Tissue Engineering: A Focus on Silk Fibroin. Materials.

[5] Gallo A.L., Pollini M., Paladini F. (2018). A combined approach for the development of novel sutures with antibacterial and regenerative properties: The role of silver and silk sericin functionalization. J. Mater. Sci. Mater. Med..

[6] Pollini M., Paladini F. (2024). The Emerging Role of Silk Fibroin for the Development of Novel Drug Delivery Systems. Biomimetics.

[7] Kim J.Y., Kim S.G., Garagiola U. (2023). Relevant Properties and Potential Applications of Sericin in Bone Regeneration. Curr. Issues Mol. Biol..

[8] Hassan M.A., Basha A.A., Eraky M., Abbas E., El-Samad L.M. (2024). Advancements in silk fibroin and silk sericin-based biomaterial applications for cancer therapy and wound dressing formulation: A comprehensive review. Int. J. Pharm..

[9] Panico A., Paladini F., Pollini M. (2019). Development of regenerative and flexible fibroin-based wound dressings. J. Biomed. Mater. Res. B Appl. Biomater..

[10] Raho R., Nguyen N.Y., Zhang N., Jiang W., Sannino A., Liu H., Pollini M., Paladini F. (2020). Photo-assisted green synthesis of silver doped silk fibroin/carboxymethyl cellulose nanocomposite hydrogels for biomedical applications. Mater. Sci. Eng. C Mater. Biol. Appl..

[11] Kunz R.I., Brancalhão R.M., Ribeiro L.F., Natali M.R. (2016). Silkworm Sericin: Properties and Biomedical Applications. Biomed. Res. Int..

[12] Lamboni L., Gauthier M., Yang G., Wang Q. (2015). Silk sericin: A versatile material for tissue engineering and drug delivery. Biotechnol. Adv..

[13] Li Y., Wu T., Zhang G., Fang A., Li Y., Wang S., Yan H., Liang P., Lian J., Zhang Y. (2023). A native sericin wound dressing spun directly from silkworms enhances wound healing. Colloids Surf. B Biointerfaces.

[14] Prakash M., Mathikere Naganna C., Radhakrishnan V., Somayaji P., Sabu L. (2024). Therapeutic potential of silkworm sericin in wound healing applications. Wound Repair Regen..

[15] Mazurek Ł., Rybka M., Jurak J., Frankowski J., Konop M. (2024). Silk Sericin and Its Effect on Skin Wound Healing: A State of the Art. Macromol. Biosci..

[16] Shitole M., Dugam S., Tade R., Nangare S. (2020). Pharmaceutical applications of silk sericin. Ann. Pharm. Fr..

[17] Das G., Shin H.S., Campos E.V.R., Singh B., Patra J.K., Fraceto L.F., Kim H.-J., Shin H.-S. (2021). Sericin based nanoformulations: A comprehensive review on molecular mechanisms of interaction with organisms to biological applications. J. Nanobiotechnol..

[18] Jo Y.-Y., Kweon H., Oh J.-H. (2020). Sericin for Tissue Engineering. Appl. Sci..

[19] Liu J., Shi L., Deng Y., Zou M., Cai B., Song Y., Wang Z., Wang L. (2022). Silk sericin-based materials for biomedical applications. Biomaterials.

[20] Dragojlov I., Aad R., Ami D., Mangiagalli M., Natalello A., Vesentini S. (2025). Silk Sericin-Based Electrospun Nanofibers Forming Films for Cosmetic Applications: Preparation, Characterization, and Efficacy Evaluation. Molecules.

[21] Tengattini S., Orlandi G., Perteghella S., Bari E., Amadio M., Calleri E., Massolini G., Torre M.L., Temporini C. (2020). Chromatographic profiling of silk sericin for biomedical and cosmetic use by complementary hydrophylic, reversed phase and size exclusion chromatographic methods. J. Pharm. Biomed. Anal..

[22] Takasu Y., Yamada H., Tsubouchi K. (2002). Isolation of Three Main Sericin Components from the Cocoon of the Silkworm, *Bombyx mori*. Biosci. Biotechnol. Biochem..

[23] Kundu S.C., Dash B.C., Dash R., Kaplan D.L. (2008). Natural protective glue protein, sericin bioengineered by silkworms: Potential for biomedical and biotechnological applications. Prog. Polym. Sci..

[24] Barajas-Gamboa J.A., Serpa-Guerra A.M., Restrepo-Osorio A., Álvarez-López C. (2016). Sericin applications: A globular silk protein. Ing. Compet..

[25] Kanoujia J., Faizan M., Parashar P., Singh N., Saraf S.A. (2021). Curcumin loaded sericin nanoparticles: Assessment for biomedical application. Food Hydrocoll. Health.

[26] Arango M.C., Montoya Y., Peresin M.S., Bustamante J., Álvarez-López C. (2020). Silk sericin as a biomaterial for tissue engineering: A review. Int. J. Polym. Mater. Polym. Biomater..

[27] Mathew S.S., Maria H.J., Allardyce B.J., Rajkhowa R., Thomas S. (2024). Waste to Wealth: Exploring the Versatile Prospects of Discarded Silk Sericin. ACS Sustain. Chem. Eng..

[28] Seo S.-J., Das G., Shin H.-S., Patra J.K. (2023). Silk Sericin Protein Materials: Characteristics and Applications in Food-Sector Industries. Int. J. Mol. Sci..

[29] Bascou R., Hardouin J., Ben Mlouka M.A., Guénin E., Nesterenko A. (2022). Detailed investigation on new chemical-free methods for silk sericin extraction. Mater. Today Commun..

[30] Züge L.C.B., Silva V.R., Hamerski F., Ribani M., Gimenes M.L., Scheer A.P. (2015). Emulsifying Properties of Sericin Obtained from Hot Water Degumming Process. J. Food Process Eng..

[31] Wang W., Pan Y., Gong K., Zhou Q., Zhang T., Li Q. (2019). A comparative study of ultrasonic degumming of silk sericin using citric acid, sodium carbonate and papain. Color. Technol..

[32] Capar G., Aygun S.S., Gecit M.R. (2008). Treatment of silk production wastewaters by membrane processes for sericin recovery. J. Membr. Sci..

[33] Capar G., Pilevneli T., Yetis U., Dilek F.B. (2022). Life cycle assessment of sericin recovery from silk degumming wastewaters. Sustain. Chem. Pharm..

[34] Wang F., Cao T.T., Zhang Y.Q. (2015). Effect of silk protein surfactant on silk degumming and its properties. Mater. Sci. Eng. C Mater. Biol. Appl..

[35] Capar G., Pilevneli T. (2024). Cost-effective process development for sericin recovery from silk degumming wastewater. Sustain. Chem. Pharm..

[36] Silva A.S., Costa E.C., Reis S., Spencer C., Calhelha R.C., Miguel S.P., Ribeiro M.P., Barros L., Vaz J.A., Coutinho P. (2022). Silk Sericin: A Promising Sustainable Biomaterial for Biomedical and Pharmaceutical Applications. Polymers.

[37] https://www.sigmaaldrich.com/IT/it/search/sericin?focus=products&page=1&perpage=30&sort=relevance&term=sericin&type=product.

[38] Gulrajani M.L., Purwar R., Prasad R.K., Joshi M. (2009). Studies on structural and functional properties of sericin recovered from silk degumming liquor by membrane technology. J. Appl. Polym. Sci..

[39] Aad R., Dragojlov I., Vesentini S. (2024). Sericin Protein: Structure, Properties, and Applications. J. Funct. Biomater..

[40] Aramwit P., Kanokpanont S., Nakpheng T., Srichana T. (2010). The Effect of Sericin from Various Extraction Methods on Cell Viability and Collagen Production. Int. J. Mol. Sci..

[41] Ghaffari-Bohlouli P., Jafari H., Taebnia N., Abedi A., Amirsadeghi A., Niknezhad S.V., Alimoradi H., Jafarzadeh S., Mirzaei M., Nie L. (2023). Protein by-products: Composition, extraction, and biomedical applications. Crit. Rev. Food Sci. Nutr..

[42] Chirila T.V., Suzuki S., McKirdy N.C. (2016). Further development of silk sericin as a biomaterial: Comparative investigation of the procedures for its isolation from Bombyx mori silk cocoons. Prog. Biomater..

[43] Da Silva T.L., da Silva Junior A.C., Ribani M., Vieira M.G.A., Gimenes M.L., Da Silva M.G.C. (2014). Evaluation of molecular weight distribution of sericin in solutions concentrated via precipitation by ethanol and precipitation by freezing/thawing. Chem. Eng. Trans..

[44] Pan M., Jin Y., Ye Y., Jiang W., Zhu L., Lu W. (2024). An efficient and eco-friendly method for removing sericin using microwave-assisted steam degumming. Environ. Technol. Innov..

[45] Capar G., Aygun S.S., Gecit M.R. (2009). Separation of sericin from fatty acids towards its recovery from silk degumming wastewaters. J. Membr. Sci..

[46] Li H., Shi W., Wang W., Zhu H. (2015). The extraction of sericin protein from silk reeling wastewater by hollow fiber nanofiltration membrane integrated process. Sep. Purif. Technol..

[47] Wu J.-H., Wang Z., Xu S.-Y. (2007). Preparation and characterization of sericin powder extracted from silk industry wastewater. Food Chem..

[48] Rueda Mira S., Quiceno N.J., Arango M.C., Tokarev A., Peresin M.S., Alexeev V.L., Álvarez-López C. (2024). Silk Sericin Films from Concentrated Aqueous Solutions: Processing Routes, Structure, and Properties. Polym. Sci. Ser. A.

[49] Wang W.-H., Lin W.-S., Shih C.-H., Chen C.-Y., Kuo S.-H., Li W.-L., Lin Y.-S. (2021). Functionality of Silk Cocoon (*Bombyx mori* L.) Sericin Extracts Obtained through High-Temperature Hydrothermal Method. Materials.

[50] Silva V.R., Ribani M., Gimenes M.L., Scheer A.P. (2012). Sericin extraction from silkworm cocoons. Procedia Eng..

